# E-Liquid Autofluorescence can be used as a Marker of Vaping Deposition and Third-Hand Vape Exposure

**DOI:** 10.1038/s41598-017-07862-w

**Published:** 2017-08-07

**Authors:** Eric S. Davis, M. Flori Sassano, Henry Goodell, Robert Tarran

**Affiliations:** 1 Marsico Lung Institute/Cystic Fibrosis Research Center, University of North Carolina, Chapel Hill, NC USA; 2 Department of Biomedical Engineering, University of North Carolina, Chapel Hill, NC USA; 3 Department of Cell Biology & Physiology, University of North Carolina, Chapel Hill, NC USA

## Abstract

In the past 5 years, e-cigarette use has been increasing rapidly, particularly in youth and young adults. Due to the novelty of e-cigarettes (e-cigs) and e-cigarette liquids (e-liquids), research on their chemo-physical properties is still in its infancy. Here, we describe a previously unknown and potentially useful property of e-liquids, namely their autofluorescence. We performed an emission scan at 9 excitation wavelengths common to fluorescent microscopy and found (i) that autofluorescence differs widely between e-liquids, (ii) that e-liquids are most fluorescent in the UV range (between 350 and 405 nm) and (iii) fluorescence intensity wanes as the emission wavelength increases. Furthermore, we used the autofluorescence of e-liquids as a marker for tracking e-cig aerosol deposition in the laboratory. Using linear regression analysis, we were able to quantify the deposition of a “vaped” e-liquid onto hard surfaces. Using this technique, we found that every 70 mL puff of an e-cigarette deposited 0.019% e-liquid (v/v) in a controlled environment. Finally, we vaped a surface in the laboratory and used our method to detect e-cig aerosol third-hand exposure. In conclusion, our data suggest that e-cigarette autofluorescence can be used as a marker of e-cigarette deposition.

## Introduction

Electronic cigarettes (E-cigs) differ from conventional cigarettes in that they do not contain combustible tobacco. Instead, e-liquids are drawn and heated over a battery-operated coil during inhalation to deliver aerosolized nicotine in a liquid vehicle (e-liquid) to the lungs. E-cig users are a fast-growing subset of nicotine users who are described as “vapers” rather than smokers, since e-cigs heat but do not burn e-liquids to generate aerosols. E-liquids contain a propylene glycol/vegetable glycerin (PG/VG) vehicle, nicotine (0–36 mg/ml) and chemical constituents for flavoring. Currently, over 7,700 different e-liquids are commercially available. Whilst first generation e-cigs, dubbed “cigalikes”, were constrained to look like cigarettes and poorly delivered nicotine to the blood^1–4^, second- and third-generation e-cigs contain a refillable tank to which the e-liquid is added, a battery-powered atomizer that generates the aerosol from the e-liquid and a mouthpiece that collects and delivers the aerosol. These newer devices are much more efficient at delivering nicotine and plasma nicotine levels comparable to what is seen with conventional tobacco smoking have now been observed^1, 4–6^. It has recently been reported that e-liquids generate relatively small size (107–165 nm and 165–255 nm, for number and volume metrics, respectively^7^) hygroscopic particles when aerosolized/vaped^8, 9^. However, since e-cigs have only been available for the last ~5 years, relatively little is known about their physicochemical properties.

Autofluorescence is the emission of longer wavelength light from biological and chemical entities after excitation in the absence of fluorescent dyes^10^. Common biological examples of autofluorescence include amino acids such as tyrosine and tryptophan, nicotinamide adenine dinucleotide and chlorophyll^11, 12^. Additionally, many polycyclic aromatic hydrocarbons that contain benzene rings are also autofluorescent including naphthalene. Indeed, benzene, naphthalene and other polycyclic aromatic hydrocarbons are present in tobacco smoke and contribute to the autofluorescence of the tar phase^13–15^. Since some flavors contained in e-liquids have benzene rings including cinnamaldehyde and vanillin^16, 17^, we hypothesized that e-liquids might be autofluorescent. We therefore tested 266 commercially available e-liquids for autofluorescence and found that many e-liquids are indeed autofluorescent. We also found that this autofluorescence can be used as a novel marker of e-liquid vapor deposition in the laboratory, and it may serve as a marker of third-hand smoke exposure.

## Results

**Figure 1:**
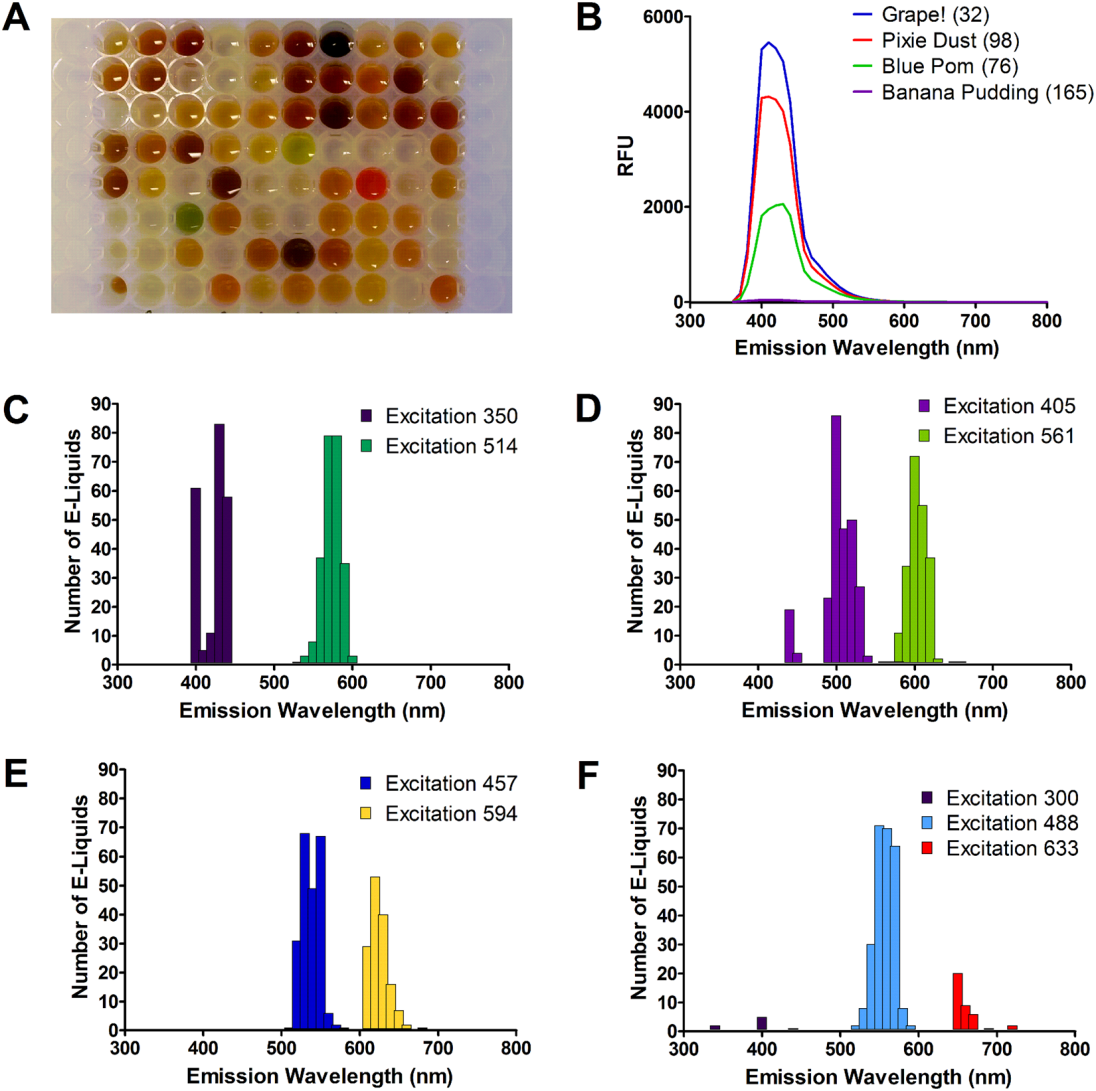
Distribution and relative intensity of e-liquid autofluorescence. 266 e-liquids were screened for autofluorescence at 9 excitation wavelengths. The signal after background subtraction was used to determine the wavelength of peak emission at each excitation wavelength. (**A**) Representative picture showing the diversity in e-liquid appearance/color. (**B**) Representative emission spectra for 4 e-liquids to illustrate differences in signal intensity at excitation/emission of 350/410 nm at a gain of 100. (**C**–**F**) The distribution of peak emission for excitation wavelengths of (**C**) 350 and 514 nm, (**D**) 405 and 561 nm, (**E**) 457 and 594 nm, (**F**) 300, 488, and 633 nm.

**Figure 2:**
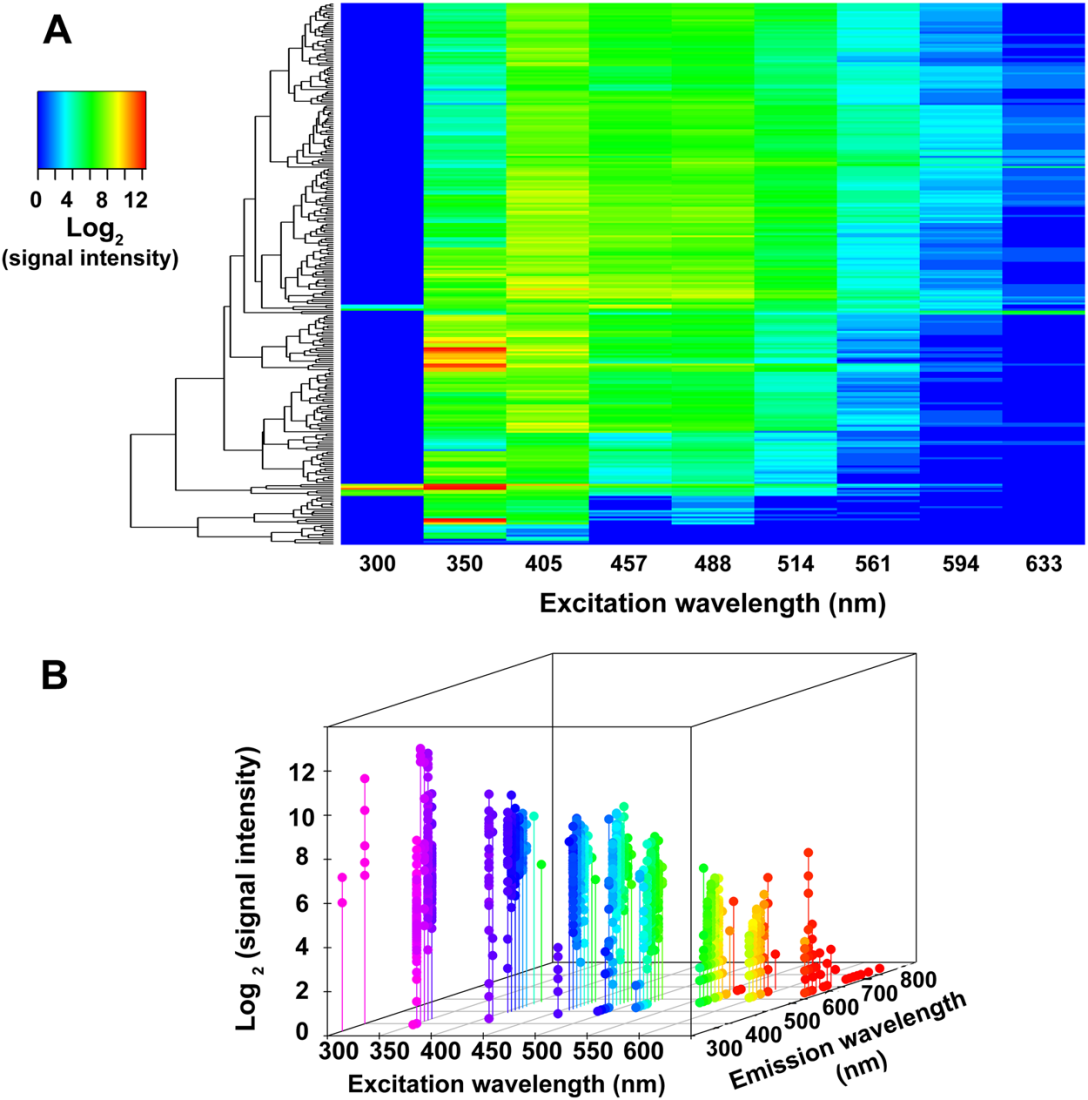
E-liquids have unique autofluorescence profiles. (**A**) Heatmap of Log_2_ (signal intensity) for all e-liquids, clustered by similarity in autofluorescent profile. (**B**) 3D scatterplot showing the emission wavelength and Log_2_ (signal intensity) for all e-liquids.

E-liquids are extremely diverse in appearance and vary from being colorless to brown, yellow, green or red (Fig. 1A). Accordingly, to look for autofluorescence, we performed emission scans on 266 e-liquids in the 384 well format. For excitation, we chose wavelengths common to fluorescent microscopy (i.e. 300, 350, 405, 457, 488, 514, 561, 594 and 633 nm). We then performed emission scans using a Tecan Infinite Pro plate reader in monochromator mode. Typical emission scans for 4 e-liquids (*Grape!, Banana Pudding*, *Blue Pom* and *Pixie Dust*) excited at 350 nm are shown in Fig. 1B. We then reanalyzed the same batches of e-liquids 4 months later. *Grape!, Blue Pom* and *Pixie Dust* increased fluorescence by 16%, 36% and 37% respectively, suggesting that this effect is extremely durable. In contrast, *Banana Pudding* had relatively little autofluorescence. We then looked at the distribution of peak fluorescence for all 266 e-liquids (Fig. 1C–F) at the different excitation wavelengths. At 350 nm excitation, emission was bimodal and ~60–80 e-liquids had peak excitations each at 400, 430 and 440 nm, with less excitation at 410 nm. Another UV wavelength tested (405 nm) was also bimodal (Fig. 1D) whilst the visible light spectra (488–594 nm) were more Gaussian in their distribution (Fig. 1C–F). In contrast, e-liquids emitted poorly in the red (633 nm) range (Fig. 1F). In addition to having a variety of emission peaks, the emission intensity also varied irrespective of the excitation wavelength. For illustrative purposes, we generated a heatmap of the emission intensities of all e-liquids (Fig. 2A) and a 3D-scatterplot showing where and how strongly each e-liquid fluoresces at a given excitation wavelength (Fig. 2B). At an excitation of 300 nm, few e-liquids were fluorescent, but those that were had strong emission peaks (Figs 1 F and 2). In general, most e-liquids were more autofluorescent at the excitations wavelengths of 300 and 405 nm. For example, the most autofluorescent e-liquid we screened (*Grape!*) was 340 times more autofluorescent than the PG/VG e-liquid vehicle at these wavelengths. In contrast, only a few e-liquids, including “*I Love Donuts”* were more autofluorescent in the far red range (Fig. 2A and Supplement S1 for raw data).

**Figure 3:**
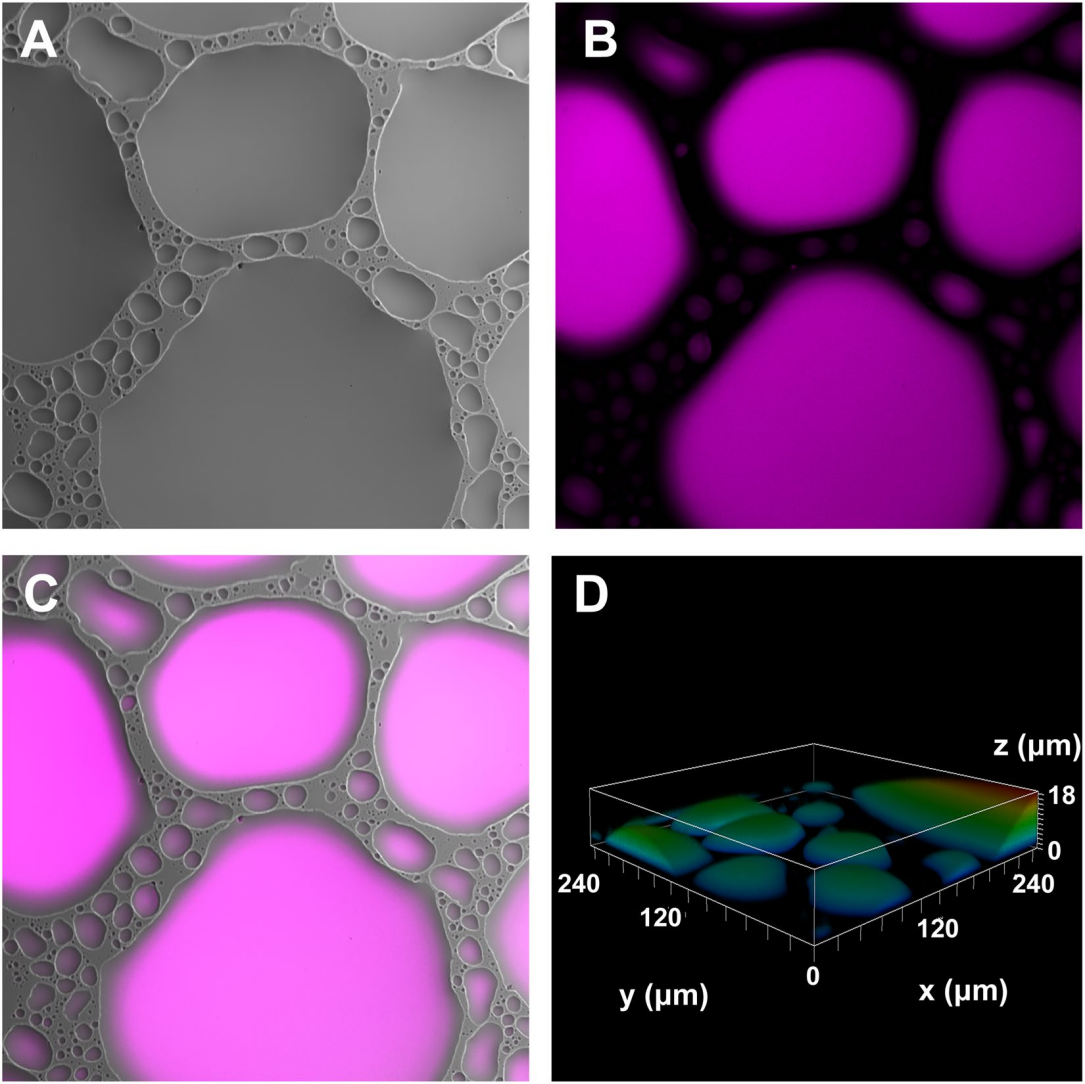
Autofluorescence as a marker of e-liquid deposition. 25 × 70 mL puffs of *Grape Soda* generated at 40 W were vaped over a glass coverslip and imaged. (**A**) Transmitted light image showing textured e-liquid deposition. (**B**) XY-confocal image of the same e-liquid, following excitation at 405 nm. (**C**) Merged images. (**D**) 3D rendering of e-liquid deposition using ~60 z-stack images.

To visually confirm e-liquid deposition, we acquired images by XY-confocal microscopy after vaping 25 puffs of *Grape Soda* onto a glass coverslip. When excited at 405 nm, droplet sized particles of *Grape Soda* were clearly visible, which could be seen in both the fluorescence and visible light images (Fig. 3A–C). We then generated a Z-stack of ~60 images which were rendered into 3D using Leica LAS Software (Fig. 3D). This image suggested that vaped *Grape Soda* was deposited as both large and small droplets. Furthermore, the imaging confirmed that the autofluorescent properties of the e-liquids were maintained after heating/aerosolizing.

**Figure 4:**
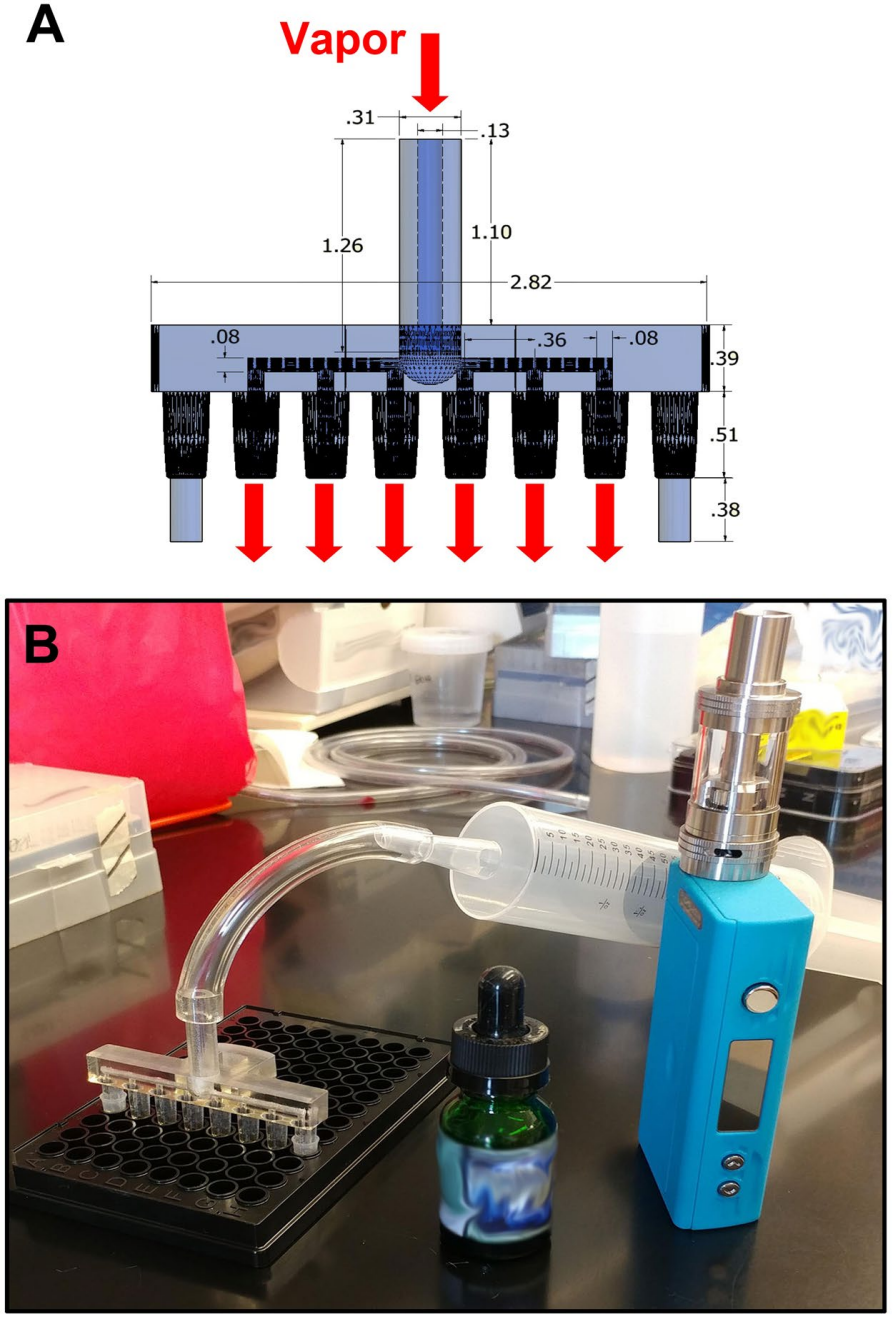
3D printed, 6-channel manifold used to “vape” 6 wells per plate simultaneously. The adaptor was fabricated using a biocompatible Med610 resin and was connected to a third-generation mod to allows for “vaping” of 96 well plates. (**A**) Autocad-generated image, with dimensions given in inches. (**B**) Photograph of actual manifold next to 96 well plate and Sigelei FuChai 200W-TC device with a Crown stainless steel subtank.

**Figure 5:**
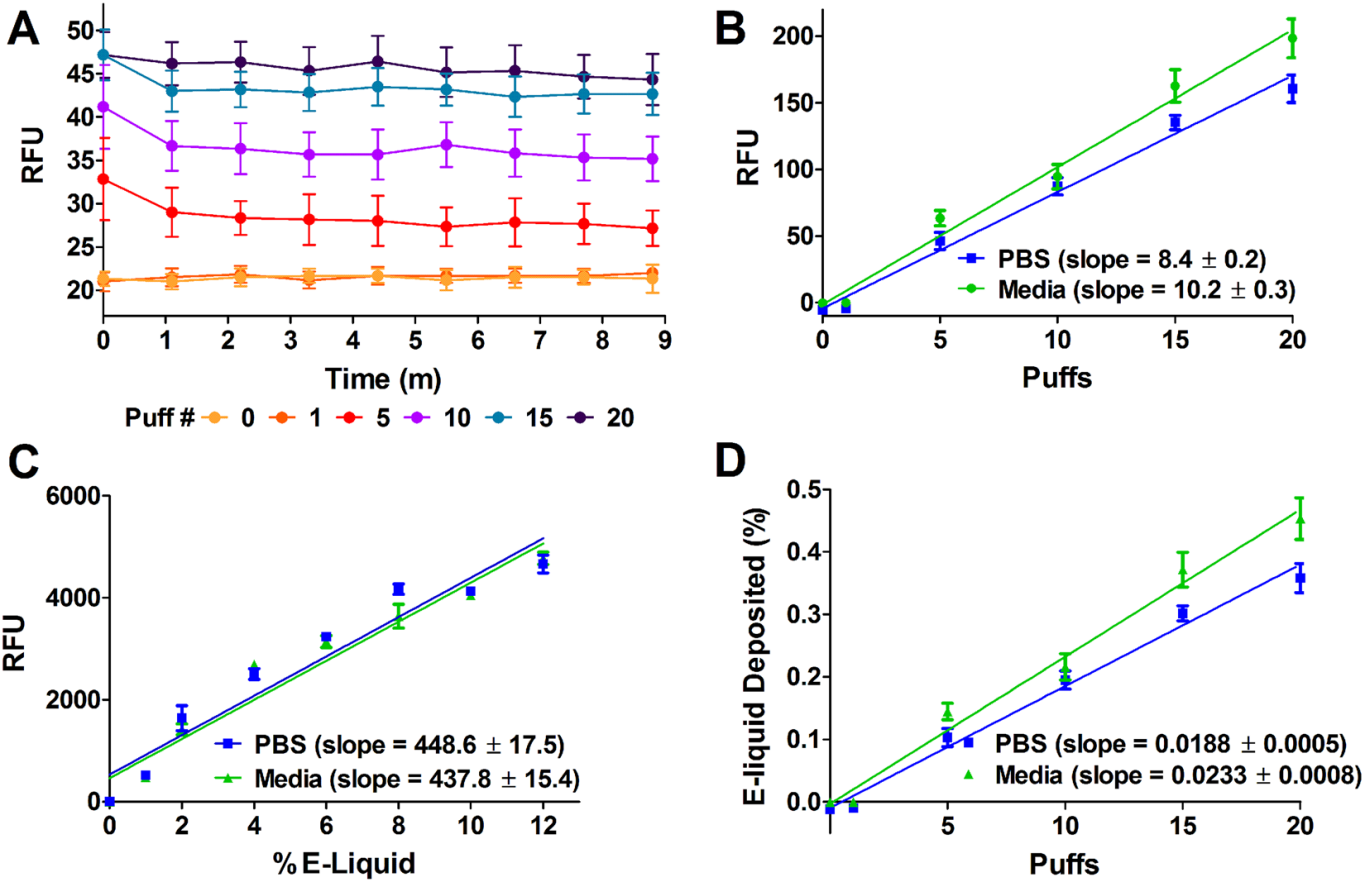
E-liquid deposition quantification by autofluorescence. *Grape Soda* e-liquid was vaped into PBS or media using 70 mL puffs generated at 40 W. (**A**) Fluorescence intensity did not change significantly with deposition after 9 minutes (n = 6). (**B**) Fluorescence intensity was proportional to number of puffs. Slopes of regression line were 8.4 ± 0.2 (R^2^ = 0.94) and 10.2 ± 0.3 (R^2^ = 0.92) for PBS and media, respectively (n = 6). (**C**) Fluorescence intensity was proportional to e-liquid concentration. Slopes of regression line were 448.6 ± 17.5 (R^2^ = 0.92) and 437.8 ± 15.4 (R^2^ = 0.94) for PBS and media, respectively (n = 6). (**D**) E-liquid deposition (% v/v) was directly proportional to the number of puffs. Slopes of regression line (y-intercept constrained to 0) were 0.0188 ± 0.0005 (R^2^ = 0.94) and 0.0233 ± 0.0008 (R^2^ = 0.92) for PBS and media, respectively (n = 6).

Since vaped e-liquids were autofluorescent, we next tested whether this phenomenon could be used to measure e-liquid deposition. To do this, we used a 3D printed, 6-channel manifold to vape up to 20 puffs of e-liquid into 100 µL of either PBS or cell culture media into 6 wells of a 96-well plate (Fig. 4). We then measured fluorescence every minute for 10 minutes with a plate reader at 405/440 nm (excitation/emission) after each set of puffs to test whether fluorescence changed over time (Fig. 5A). Since fluorescence emission was stable, we used this approach to test whether autofluorescence could be used as a marker of deposition. Our data indicated that *Grape Soda* autofluorescence after vaping correlated with autofluorescence of the e-liquid before it was vaped and this fluorescence increased linearly with increased puff number (slope in PBS = 8.4 ± 0.2, slope in media = 10.2 ± 0.3; Fig. 5B). To quantify the deposition delivered per puff we created a standard curve by directly adding the same e-liquid in PBS and Media at known concentrations ranging from 0 to 12% (v/v). Again, there was a linear relationship (slope in PBS = 448.6 ± 17.5, slope in media = 437.8 ± 15.4) between the percentage of e-liquid diluted and autofluorescence (Fig. 5C). Using linear regression analysis (Fig. 5D), we were able to convert RFU fluorescence values to percent e-liquid deposited by volume, thus allowing direct conversion between the number of e-liquid puffs and the percentage deposition (Fig. 5D). As such, we determined the percentage of e-liquid deposited per puff to be 0.019%.

**Figure 6:**
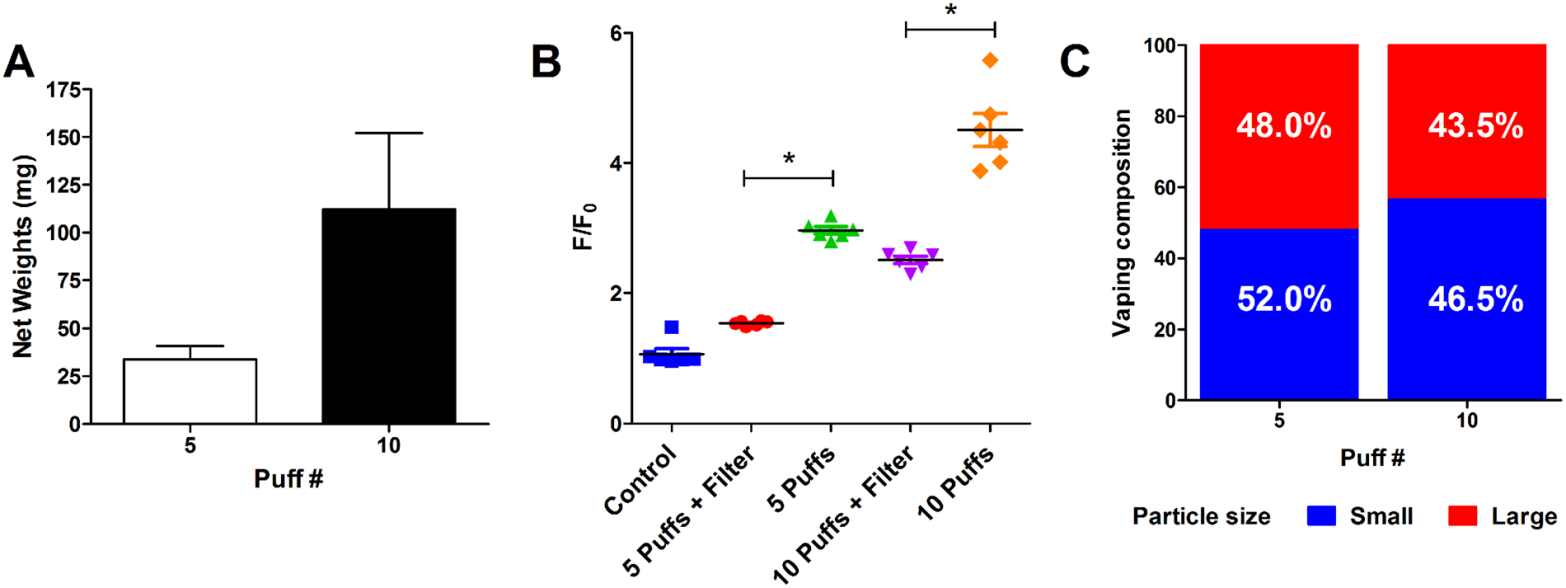
Quantification of e-cigarettes produced vapor and aerosol phases. E-liquid flavor *Grape!* was vaped 5 and 10 times (70 mL puffs, 100 W) with and without EPM-2000 filter pads into PBS. (**A**) Filter pad weight increased with puff number, trapping the larger aerosolized e-liquid particles (n = 3). (**B**) Fluorescence decreased in the presence of a filter compared to covered controls (n = 6). (**C**) The fluorescence detected from filtered puffs compared to unfiltered puffs of e-liquid vape as indicated (n = 6).

The exact composition of e-liquid effluence is controversial^18, 19^. This is further complicated by the “vaping” terminology. According to recent publications, e-liquid effluence is an aerosol composed of submicron and nanoparticles^18, 19^. Therefore, we used the autofluorescence of vaped e-liquids to test whether we could detect particle deposition. Accordingly, we vaped 5 or 10 puffs of *Grape Soda* e-liquid into 96 well plates, either directly, or with a Whatman EPM-2000 filter pad (pore size of 0.2 µm) inserted in the line to filter aerosol particles. Using a microbalance, we were able to detect significant deposition onto the filter pads after 5 and 10 puffs (Fig. 6A) and both 5 and 10 puffs resulted in significant autofluorescence, as detected by the plate reader (Fig. 6B). For both 5 and 10 puffs, the presence of the EPM-2000 filter pad reduced autofluorescence by 52.0% ± 1.80% and 56.5% ± 6.0% respectively but did not abolish it, suggesting that the autofluorescence can be used to detect multiple-sized particles (Fig. 6C). Therefore, we conclude that e-cigarette “vape” is composed of roughly 50% small and 50% large particle aerosol.

**Figure 7:**
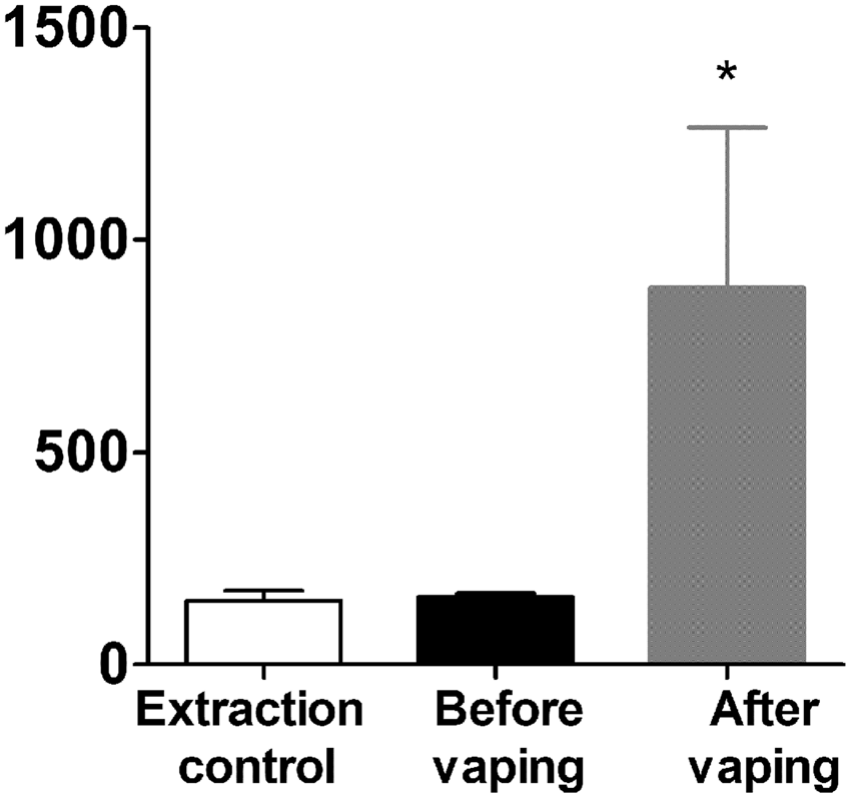
Detection of e-cigarettes third-hand smoking deposition. (**A**) E-liquid flavor *Grape!* was vaped 10 times (70 mL puffs, 100 W) onto a sheet of aluminum foil and deposition was collected and extracted in DMSO. Fluorescence (ex: 350 nm, em: 410 nm) after vaping was higher than before vaping (*p < 0.05).

Third-hand exposure occurs when products are smoked (or vaped) and deposited on surfaces where they can be taken up transdermally, which is a significant health concern^20–23^. As a proof of concept, we decided to test whether autofluorescence of e-cigarette vape could be detected on exposed surfaces. To simulate a third-hand exposure scenario, we vaped *Grape!* e-liquid onto a clean sheet of aluminum foil and used quartered EPM-2000 filter pads to swipe the surface. After extraction in DMSO, we were able to detect significantly higher levels of fluorescence with the plate reader after vaping compared to vehicle and pre-vaping controls (Fig. 7), suggesting that our autofluorescence technique could be used to detect third-hand e-cigarette vape exposure.

## Discussion

In this paper, we have demonstrated that e-liquids are highly autofluorescent. Indeed, the emission scans revealed a great deal of heterogeneity between e-liquids with respect to autofluorescence. That is, both signal strength and distribution of peak emission varied considerably from one e-liquid to another (Figs 1 and 2; Supplement Table S1). The majority of e-liquids appeared to have a unique fluorescence profile. However, distinct trends were discernable: most e-liquids were autofluorescent in the UV spectrum, exciting between 300 and 405 nm and emitting between 340 and 550 nm. The opposite was true of the deep red region, where peak emission signal strength tended to diminish. The most strongly autofluorescent e-liquids (most notably “*Grape!”* and *“Grape Soda”*) fluoresced in this region, but had the highest signal strength at an excitation wavelength of 350 nm (Figs 1 and 2). We found that the vehicle (PG/VG) was only very weakly autofluorescent (Table S1). Furthermore, nicotine did not affect autofluorescence. For example, *Kola No Nicotine* was actually more autofluorescent than *Kola* (with nicotine) and *Bada Bing!*, which was nicotine-free, was extremely autofluorescent (Table S1). Likely, as stated in the introduction, organic compounds with benzene rings contributed to the autofluorescence. Whilst we could still detect significant autofluorescence after vaping (Figs 3 and 5), it is possible that pyrolysis may alter the chemical properties and autofluorescence of some of these organic compounds, especially the volatiles and semi-volatiles. Thus, further studies will be required to determine whether pyrolysis affects autofluorescence. Of note, we found that autofluorescence increased by up to 37% when a batch of e-liquids were stored for 4 months and remeasured using exactly the same settings. Whether this was due to a change in the e-liquid chemical composition or other extrinsic factors remains to be determined. Importantly, these experiments demonstrate that this is an extremely durable effect.

We propose that these novel properties will be useful in studying e-liquids in the laboratory and must also be taken into consideration to avoid artifactual readings. For example, in assays that use fluorescent dyes to stain cells, e-liquid autofluorescence has the potential to interfere and give artificially high readings^24^. However, we propose that autofluorescence can be an advantage that can be utilized as a marker of e-cig vape deposition. Since we confirmed by confocal microscopy that autofluorescence of e-liquids was conserved after vaping and subsequent deposition on surfaces (Fig. 3), we were able to translate and quantify the relationship between puffs from the e-cigarette, and the amount of resulting e-liquid that is deposited. In general, for every 70 ml puff from our vaping apparatus, we are able to deliver an e-cig vape concentration equal to a 0.019% e-liquid (vol/vol) concentration (Fig. 5). This meant that 20 puffs from our vaping system delivered a concentration equivalent to ~0.38% e-liquid (vol/vvol) per well in 100 μl of PBS or media in a 96 well plate. As a caveat, these exact numbers were only applicable to the vaping system that we used. However, given that autofluorescence does not appear to be influenced by vaping, this parameter should be measurable and reproducible with different device types and different puff parameters, although less efficient models would be predicted to yield less vapor per puff and hence less autofluorescence.

This autofluorescence may also be influenced by the sensitivity of the system used to record it. For example, despite good curve fitting, the amount of fluorescence was less after vaping than after direct e-liquid deposition (see Fig. 5B and C). Here, we kept the gain the same so that they could be directly compared. However, when measuring e-liquid deposition after vaping, the gain and/or lamp intensity could be increased as needed for greater sensitivity. Furthermore, a similar approach could be used for other experimental setups and such an approach will allow researchers to more accurately determine the amount of e-liquid that is being deposited when they use e-cigarette puffing in their assays.

There has been some debate about the physico-chemical properties of e-liquids after they have been vaped, including whether or not the effluent is in a vapor or an aerosol form^25, 26^. An aerosol is defined as a colloidal suspension of particles that is dispersed in air or gas while vapor is defined as the gas phase of a substance. Recent publications have shown that e-liquids are composed of different sized sub-micron and nanoparticles^18, 19^. When e-liquid aerosol is inhaled, potentially toxic chemicals in or on these particles are deposited in the respiratory tract^18^. These particles pose a higher potential risk than larger aerosol particles, due to their ability to evade innate respiratory clearing mechanisms^27^. Furthermore, submicron particles are deposited throughout the airways and retained deeper in the respiratory tract than large aerosol particles, where they have the potential to do harm^28^. Due to the recent advent of vaping, little information is available in the literature about e-liquids after they have been vaped. Here, we have demonstrated that approximately 50% of the output from e-cig vaping are able to penetrate the filter pad and roughly 50% is trapped by the filter pad (Fig. 6). While more work will be required to determine the particle size and composition under a variety of different conditions, here we have shown that both filtered and unfiltered e-cig vape are autofluorescent and detectable using a standard plate reader.

One route of exposure to e-cigs that has not been explored is third-hand exposure. Third-hand smoke exposure for tobacco products is a potential exposure route to tobacco smoke that is often overlooked, and can be harmful to children’s health^29^. Third-hand smoke pollutants that are present in dust, air and on surfaces have been shown to persist in an indoor environment for several months after smoking has ceased, even after cleaning^30^. This provides a potential route of exposure to some of over 4,000 harmful chemicals present in tobacco smoke^29^. We used autofluorescence as a marker to look for potential third-hand e-cig exposure on surfaces. Here, we demonstrated that autofluorescence was detectable after an EPM-2000 filter pad was swiped across a vaped surface, the residue extracted in DMSO and measured using a plate reader. While it is possible to use gas chromatography-mass spectrometry (GC-MS) to detect nicotine in the homes of vapers as a measure of third-hand vape (THV) exposure^31^, GC-MS devices are expensive and require specialized personnel to run. Whilst our data concurs with theirs and confirms the potential for third-hand vape exposure, one advantage of our method is that the apparatus required to read the autofluorescence (in this case a plate reader) is significantly cheaper to purchase and typically more ubiquitous in the laboratory than mass spectrometry machines. Furthermore, since we performed full spectral scans to characterize the e-liquids, using ~350 or ~405 nm excitation, and the appropriate emission (e.g. 440 ± 15 nm), e-liquid autofluorescence could also be determined by fluorescence microscopy. The ability to detect third-hand exposure may be useful for monitoring exposure in public places such as restaurants and movie theaters where the general public including children and pregnant mothers could be unintentionally exposed to third-hand vaping. However, as a caveat, since tobacco smoke is also highly autofluorescent, care would be needed to ensure that cross-contamination did not occur.

In conclusion, our study provides novel information about an unexplored phenomenon – namely the autofluorescence of both neat and vaped e-liquids. We suggest that this property has many applications including detecting the presence of e-liquids in biological assays, quantification of deposition, as well as detection of third-hand exposure on external surfaces. Indeed, we propose that this autofluorescence will be useful in the laboratory as a marker of deposition both to cell cultures and to hard surfaces, that can be used to assess first, second and third hand exposures. Furthermore, e-liquid autofluorescence may constitute a novel biomarker of exposure, for example in induced sputum or nasal lavage. Due to the novelty of e-liquids and e-cigarette devices, further exploration of atomization and vapor/aerosol generation may help inform researchers on the nature of their deposition and dispersion. Importantly, our data suggest that vaped e-liquids pose a previously under recognized risk to public health, namely that of third-hand vape exposure and we propose that further research will need to be performed in order to fully understand the implications of these findings.

## Methods

### Flavored E-cigarette Liquids (e-liquids)

We purchased E-liquids from a variety of commercial sources, as indicated in Table S2. The e-liquids were selected to be diverse in both composition and flavor. They also represented a variety of nicotine concentrations, ranging from 0 to 36 mg/mL, and a variety of ratios of propylene glycol (PG) to vegetable glycerin (VG) from 30:70 to 100% VG.

### Chemicals, Reagents, and Materials

Propylene glycol, vegetable glycerin and DMSO were purchased from Sigma-Aldrich. FluoroBrite DMEM (Media) and PBS were purchased from Thermo-Fisher. Assays were performed in Corning Costar 96 and 384 well black-walled clear bottom polystyrene plates purchased from Sigma-Aldrich. Control wells were covered by a 96 well round well silicone mat, cut into strips, purchased from Thomas Scientific. E-cig effluent was drawn into 100 mL syringes purchased from Thermo-Fisher. E-liquids were photographed in a white walled Nunc 96 well plate purchased from Thermo-Fisher.

### E-cigarette and Aerosol Generation

E-cigarette aerosols were generated using a Sigelei FuChai 200W-TC device with a Crown stainless steel subtank with a 0.25 Ω SUS316 dual coil from Uwell. Aerosols were produced by activating the e-cigarette device while drawing into a 100 mL syringe from the mouthpiece of the subtank. Based on existing e-cigarette topography^32–34^, we typically generated 70 mL puffs drawn over approximately 5 seconds and dispensed at approximately 0.84 L/min at 100 W, unless otherwise stated. To directly vape into 96 well plates, we generated a 3D printed manifold. This manifold was designed using AutoCad and was a modification of an existing manifold design (https://3dprint.nih.gov/discover/3DPX-003200) where the outer 2 channels were filled in to stabilize the manifold *in situ* during the vaping protocol. The modified manifold was fabricated using a biocompatible Med610 resin on an Objet 3D printer (Fig. 4A). The AutoCad file is available on request. These manifolds were used to simultaneously vape 6 wells per plate (Fig. 4B).

### Emission Scans

Approximately 20 µL per e-liquid of 266 different e-liquids were aliquoted directly without further dilution into individual wells of a 384 well plate. Full emission spectra were then obtained in 10 nm increments from 9 different excitation wavelengths (300, 350, 405, 457, 488, 514, 561, 594 and 633 nm) using a Tecan Infinite Pro Plate reader in monochromator mode. Emission bandwidth was 5 nm; excitation bandwidth was 2.5 nm for 300 nm and 5 nm for 350–633 nm, respectively. For each excitation wavelength, the peak fluorescence intensity of emission was determined after background subtraction using a H_2_O-filled well. All fluorescence values are reported as relative fluorescence units (RFU) unless otherwise stated.

### Confocal Microscopy

25 × 70 mL puffs of *Grape Soda* generated at 40 W were vaped onto a #1 coverslip contained in an Attofluor imaging chamber (Thermo-Fisher). Images were then acquired with the 405 nm laser line and collected at 440 ± 20 nm using a Leica SP8 confocal microscope with a 63 × 1.3 NA glycerol immersion lens. A Z-stack of ~60 images were generated and rendered into 3D using Leica LAS Software.

### Aerosol Deposition and Standard Curves

0, 5, 10, 15, or 20 puffs of e-liquids were vaped into different columns of 96 well, black plates containing 100 µL/well of either PBS or Media using the 3D printed adaptor. After each set of puffs, the plate was read approximately every minute for 10 minutes using a Tecan Infinite Pro plate reader to detect fluorescence of deposited e-liquid compared to control wells containing 100 µL of either PBS or Media covered with a 96 round well silicone mat. To generate a standard curve, e-liquids were diluted into either PBS or Media at various known concentrations ranging from 0 to 12% into 96 well plates containing 100 µL/well.

### Aerosol Filtration

Aerosolized e-liquid particles were collected using a Whatman 47 mm filter pad (EPM-2000; 0.2 μm pore size) from GE Healthcare Life Sciences and filter pad holder from ThermoFisher Scientific. Filter pads were weighed before and after being vaped with 5 or 10 puffs (see E-cigarette and Aerosol Generation section) using a Kahn analytical microbalance. E-liquids were vaped into 96 well plates with and without filter pads using the 3D printed 6-channel adaptor, compared to a covered control, and the plate was measured for fluorescence intensity using a Tecan Infinite Pro plate reader.

### Third-Hand Exposure Detection

10 puffs of an e-liquid were vaped onto a sheet of aluminum foil to simulate a third-hand exposure environment. Whatman EPM-2000 filter pads were divided into 4 equal parts and each part was swiped across the surface of the aluminum foil after vaping. To determine the background reading, additional segments of filter pads were swiped across foil before the exposure. Each filter pad segment was immersed in 500 µL of DMSO and sonicated for 15 minutes to produce e-cigarette aerosol extract. 100 µL of each sample was added to a 96 well plate and measured for fluorescence intensity compared to vehicle and pre-vaping surface swipe controls.

### Statistical Analysis

Statistical analysis was performed using GraphPad Prism 5.0 with p < 0.05 considered as significant. All values are shown as Mean ± SEM. Data were analyzed by non-parametric ANOVA. All experiments were performed on at least 3 separate occasions. Graphics for Fig. 2 were made using the statistical programming language R. In addition to base R (2016), scatterplot3d, gplots, and RColorBrewer extention packages were used.

## Electronic supplementary material

Supplemental Information
