# Supplementary material for: E-Liquid Autofluorescence can be used as a Marker of Vaping Deposition and Third-Hand Vape Exposure
Source: Sci Rep. 2017 Aug 7;7:7459. doi: 10.1038/s41598-017-07862-w (PMC5547159; doi:10.1038/s41598-017-07862-w)
Supplement: Supplementary file 1 — Supplemental Information [file 41598_2017_7862_MOESM1_ESM.pdf]

E-Liquid Autofluorescence can be used as a Marker of Vaping Deposition and Third-Hand Vape Exposure

Eric S. Davis[1], M. Flori Sassano[1], Henry Goodell[2] and Robert Tarran[1,3*]

[1]Marsico Lung Institute/Cystic Fibrosis Research Center; [2]Department of Biomedical Engineering; [3]Department of Cell Biology & Physiology; University of North Carolina, Chapel Hill, NC.

*Correspondence to: Robert Tarran
Address: Marsico Lung Institute
Marsico Hall, 125 Mason Farm Road,
University of North Carolina at Chapel Hill,
Chapel Hill, NC, 27599.
Email: robert_tarran@med.unc.edu
Contact: 919-966-7052

Table S1- Relative fluorescent intensity of e-liquids at various excitation wavelenghts. 266 e-liquids were screened for autofluorescence at 9 excitation wavelenghts in a Tecan Infinite Pro Multiplate reader using the 384-well plate format.

E-liquid name (Internal Reference Number)	Excitation wavelength (± 5 nm)								
	300	350	405	457	488	514	561	594	633
	Peak emission signal (relative fluorescent units)								
1 Leaf Tobacco (69)	0	167	121	21	29	9	3	0	0
10 Leaves Tobacco (65)	0	357	100	10	18	7	1	1	1
5 Leaves Tobacco (61)	0	230	165	44	49	16	5	1	0
55:45 PGVG (164)	0	1	16	-1	0	0	1	1	1
Adam's apple (173)	0	21	273	121	94	35	7	3	2
Adam's apple (194)	0	15	167	99	92	40	8	3	1
Admiral Berry Crunch (227)	0	37	332	183	205	91	17	5	1
Alchemy (34)	0	4325	327	77	73	26	5	2	0
Ambrosia (27)	0	17	122	104	138	52	9	3	2
Angel Lust (23)	0	20	217	82	83	42	17	7	3
Anti-Venom (215)	0	22	65	14	34	10	7	16	38
Apple (219)	0	127	332	339	353	149	19	7	2
Apple (256)	0	17	183	207	244	93	21	8	1
Apple (275)	0	-6	-1	-11	-1	0	0	0	0
Apple Candy (178)	0	163	422	198	140	56	10	3	0
Apple Candy (234)	0	53	63	36	73	20	3	2	0
Apple Cinnamon (218)	0	20	171	144	266	153	42	14	4
Apple Pie (122)	0	14	161	84	106	57	24	9	3
Appletini (175)	0	112	61	14	24	6	1	1	0
Arctic Blueberry (62)	0	224	131	23	28	9	2	2	1
Arctic Ice (191)	0	24	83	-3	2	1	0	0	0
Arctic Raspberry (78)	0	237	163	44	50	18	4	1	1
Arctic Strawberry (102)	0	404	224	91	75	26	4	1	0
Arctic Tobacco (68)	0	183	195	58	53	14	3	1	1
Artist Liquids (241)	0	84	52	3	3	0	2	1	0
Ashes to Ashes (185)	0	184	418	107	89	26	5	2	0
B&H (203)	0	90	565	317	270	68	10	4	1
Bad Monkey (195)	0	1123	462	40	70	30	7	2	1
Bada Bing! (158)	0	5236	74	4	7	2	2	1	0
Bahama Mama (45)	0	312	246	120	163	57	8	2	0
Baja Burst (186)	0	148	221	123	106	40	6	3	2
Banana (114)	0	42	163	84	83	31	7	3	0
Banana Nut Bread (288)	0	20	157	73	84	37	15	8	2
Banana Nut Bread (289)	0	14	94	20	33	13	6	0	0
Banana Nut Bread Smoothie (42)	0	21	123	63	81	37	13	5	3
Banana Pudding (166)	0	93	37	-1	5	1	2	0	0
Banana Pudding Southern Style (129)	0	20	107	46	61	31	13	6	4
Banana Pudding Southern Style (165)	0	27	163	75	96	47	12	4	1
Banana Yo! (Yogurt!) (63)	0	80	224	152	156	70	11	3	1
Barista (180)	0	33	45	8	31	10	2	1	1
Barlett Pear (49)	0	28	133	97	66	18	4	2	0
Bat Juice (249)	124	87	111	11	25	9	4	0	0
Battle Frogs (26)	0	646	162	54	46	13	2	1	0
Bavarian Creme Donut (115)	0	10	74	28	45	22	9	6	3
Behn Johnson's Nebula (168)	0	3056	358	245	257	100	15	3	2
Berry Creamy (228)	0	57	38	-2	3	0	1	1	1
Biscotti (89)	0	18	202	78	79	40	19	8	3
Black and Blue Berries (142)	0	88	409	181	104	29	3	2	2
Black Cherry (125)	0	38	197	72	87	42	16	3	0
Black Coffee (90)	0	36	136	82	112	52	21	15	4
Black Dragon (20)	0	78	117	14	22	9	2	0	0
Black Hawk (80)	0	49	256	160	135	46	10	5	2
Black Honey Tobacco (57)	0	45	330	99	63	28	11	3	2

Black Licorice (12)	0	72	178	72	69	24	4	2	1
Black Peppercorn (101)	0	115	154	47	47	14	3	2	1
Black Thorn (109)	0	63	389	200	186	59	9	3	2
Blackberry Lemonade (53)	0	131	214	97	89	31	7	2	0
Blackberry Panda (44)	0	3382	684	55	51	14	3	2	0
Blood Orange (141)	0	188	62	8	16	4	2	0	0
BLU Refilll Tobacco (270)	0	11	143	106	140	62	20	10	3
Blue Cherry (257)	0	28	192	160	185	68	16	5	1
Blue Classic Tobacco (262)	0	70	279	297	416	151	18	6	2
Blue DUDE (59)	0	177	154	61	56	18	5	2	0
Blue Mist (276)	0	41	53	11	25	11	2	0	0
Blue Moo (106)	0	23	125	64	74	31	10	4	1
Blue Pom (Pomegranate) (76)	0	2044	217	79	50	12	2	1	1
Blueberry (207)	0	34	2	-4	-1	0	1	1	0
Blueberry Cinnamon-Streusal Muffin (100)	0	18	165	81	76	31	10	3	1
Blueberry Lemonade (55)	0	1121	152	49	50	17	4	1	1
Blueberry Tobacco (39)	0	669	157	28	31	9	1	1	1
Bombies-Product X (206)	0	30	327	110	114	58	20	6	2
Briar Patch (67)	0	19	190	111	130	58	12	4	1
Brittany's Blue Blend (50)	0	991	214	78	78	28	5	2	1
Bubble Gum (56)	0	91	122	30	42	15	5	1	1
Bubbly Berry (79)	0	189	122	37	43	14	5	1	0
Butter Crunch (135)	0	86	355	190	196	77	21	8	2
Butter Pecan (220)	0	18	365	196	129	45	11	4	1
Butterscotch (112)	0	27	312	134	136	55	12	4	2
Buttery Nipple (96)	0	34	405	140	134	54	11	3	1
Cake Batter (295)	0	16	183	115	148	68	18	10	3
Candy Cane (60)	0	16	307	103	70	34	16	6	1
Candy Corn (43)	0	15	217	89	96	51	19	7	3
Cappuccino (201)	0	38	444	129	124	47	7	1	0
Cappuchino (184)	0	53	374	153	169	71	21	9	3
Captain Suckle (19)	0	19	153	55	65	31	10	3	0
Captain Zack (47)	0	21	145	76	88	42	15	5	2
Captain Zack Cigar (128)	0	61	36	1	11	3	1	1	0
Caramel (188)	0	31	160	57	70	28	5	1	1
Caramel Apple (111)	0	21	163	91	121	59	18	7	2
Caramel Corn Crunch (64)	0	22	175	124	149	61	23	9	2
Carnage (171)	0	17	113	27	40	11	2	1	0
Cat Nip (105)	0	2778	522	148	133	46	10	2	1
Chai (84)	0	21	237	104	107	57	23	7	2
Chai Latte (85)	0	37	224	99	117	60	17	5	1
Chai Tea (216)	0	13	110	96	170	124	66	7	2
Cheese cake (179)	0	20	268	188	126	42	8	3	1
Cheesecake with Graham Cracker Crust (9	0	24	165	88	104	47	15	5	1
Cherry (274)	0	51	39	-3	3	0	0	1	0
Cherry Kola (16)	0	182	143	155	122	38	8	3	2
Chicken and Waffles (37)	0	415	244	92	84	29	6	2	0
Chocolate Banana (87)	0	14	74	23	38	19	10	7	3
Chocolate Covered Raisins (54)	0	134	149	60	74	34	14	10	5
Chocolate Dipt Raspberries (104)	0	30	124	78	115	63	24	10	4
Chocolate Fudge (82)	0	14	85	38	57	28	14	7	3
Chocolate Moo (74)	0	19	101	50	71	37	16	7	3
Chocolate Pecan Fudge (95)	0	17	105	40	56	26	12	7	3
Chocolate Tobacco Heaven (126)	0	27	188	75	89	45	17	8	2
Cig (273)	0	108	390	231	206	70	11	3	0
Cinnamon (294)	0	4	124	64	54	15	3	1	1
Cinnamon Roll (15)	0	21	178	86	86	38	15	6	1
Cinnamon Roll (192)	0	17	215	130	151	63	16	8	2
Cinn-Full Pear (300)	10	108	255	473	178	41	22	7	0
Cinn-Full Pear (301)	8	104	205	344	150	43	22	8	0
Circus Guava (75)	0	22	145	89	117	58	16	8	2
City of Angels (163)	0	9	10	0	1	0	1	0	0
Classic Tobacco (136)	0	136	120	33	42	13	3	2	0
Classics Cookie Monster (229)	0	79	581	123	107	33	7	3	2
Classics Cookie Monster (230)	0	59	198	89	80	33	13	5	1

Cloud Candy (200)	0	29	240	76	68	30	7	3	0
Clove (172)	56	48	171	82	95	37	10	5	2
Clove Cigar (123)	0	56	131	59	70	30	10	3	1
Coconut Rum (107)	0	110	269	150	162	72	22	7	2
Coconut Water (72)	0	228	155	56	53	15	4	2	0
Cola (287)	0	2	2	-5	-1	0	1	0	0
Comb The Desert (199)	0	68	79	4	8	2	1	0	0
Cool Mint (124)	0	20	161	54	51	26	10	4	2
Cotton Berry (108)	0	17	116	58	80	40	14	7	3
Cotton Candy (197)	0	5	67	23	64	43	10	3	0
Crabtastic! (24)	0	144	248	103	75	22	4	1	1
Cranberry Crunch (117)	0	74	212	149	106	31	5	3	1
Cranberry Delight! (99)	0	72	130	20	22	7	1	0	1
Crispy Melon (121)	0	116	187	166	161	64	8	2	2
Cuba Libre (30)	0	463	111	193	168	52	12	4	1
DB's Dessert (58)	0	36	223	134	129	52	12	4	2
Death Flirt (28)	0	137	311	166	138	45	9	2	0
Desert Cow (22)	0	363	336	68	61	17	4	1	0
Devil Cut (170)	0	5	103	111	105	33	7	2	0
Double Espresso (137)	0	210	890	359	271	89	14	4	2
Dragon's Blood (247)	0	32	215	132	137	65	20	8	2
Dragon's Tear (246)	0	114	66	-2	5	1	1	1	0
Drunk Monkey Banana Rum Cream (209)	0	12	157	71	82	37	9	3	2
Dulce de Leche (73)	0	20	153	80	103	53	18	8	4
Energon (13)	0	770	164	16	24	9	2	2	0
Fab 5 (268)	0	132	200	133	132	43	7	2	1
French Vanilla Cinnamon Coffee (83)	0	15	157	96	104	47	17	8	3
French Vanilla Hazelnut Espresso (86)	0	30	176	71	90	45	16	6	2
Fruit Loopy (41)	0	38	285	110	93	48	19	7	2
Fruity Juice (233)	0	43	12	-5	-1	0	1	1	0
Georgia Juicy Peach (33)	0	262	78	9	18	6	3	1	0
GG (261)	0	22	134	72	79	34	10	4	1
Glazed Doughnut 18 (244)	0	87	284	211	178	61	12	3	2
Gob-Stopper (232)	0	1155	181	103	143	67	11	3	1
Grandmaster (296)	0	26	263	184	159	59	7	2	0
Grape Soda (103)	266	3671	1154	184	141	40	10	4	1
Grape! (32)	2199	5435	207	23	28	9	1	0	1
Green Gummies (159)	0	13	3	-2	0	0	0	0	1
Gummy (223)	0	24	172	88	90	41	10	4	1
Hawaiian Punch (224)	0	231	277	159	152	60	12	4	1
Heavy Hitters (205)	0	26	233	101	134	53	9	4	0
Honey Vanilla Tobacco (119)	0	27	157	78	97	49	20	7	2
Hot Cinnamon Candies (130)	0	28	67	7	19	7	3	1	1
Hot Cinnamon Candies (4)	0	10	151	94	125	70	27	7	2
I Love Donuts (226)	0	13	169	89	168	85	24	16	22
Icy Blast (81)	0	113	82	7	14	5	1	0	0
Jay's A straw From Heaven (208)	0	36	170	61	65	30	12	5	2
Key Lime Pie (91)	0	84	206	108	125	51	13	6	3
KIWI Blast (113)	0	83	156	52	77	30	5	1	1
Kola (2)	0	144	63	145	141	44	10	5	2
Kola No Nicotine (131)	0	824	134	0	8	1	1	1	0
Lemon Meringue Pie (110)	0	48	313	110	88	41	14	5	2
Lock Jaw (235)	0	209	538	104	73	19	5	1	0
Looper ANML (225)	0	107	242	116	90	23	4	1	1
Looper-ANML (237)	0	78	344	127	110	41	10	7	2
Magid Dragon (174)	0	36	412	210	47	3	2	0	0
Marc's Burro Tobacco (17)	0	22	259	191	146	47	9	2	0
Marshmallow (71)	0	19	447	139	130	69	21	7	2
Menthol (138)	0	115	73	14	24	8	1	0	1
Menthol (253)	0	131	453	382	401	155	33	12	4
Menthol (255)	0	45	201	192	252	107	28	10	2
Menthol Tobacco (10)	0	40	242	86	93	37	10	4	1
Menthol Tobacco (132)	0	156	115	22	31	10	2	1	1
Mint (269)	0	119	26	6	6	1	0	0	0
Missed Her Cookie (88)	0	33	163	81	94	44	16	7	2

Mochachino (293)	0	28	154	106	172	99	36	15	4
Mojito (160)	0	46	4	-3	0	0	0	0	1
Mt. DUDE (29)	0	208	130	93	74	23	5	2	1
Nana Banana (189)	0	110	152	76	89	37	9	2	0
NE (263)	0	51	202	186	242	84	14	5	1
Night Time's blues (231)	0	243	171	85	91	33	5	3	1
NJOY (258)	0	55	211	138	170	49	7	2	1
NJOY (259)	0	235	172	18	22	7	2	0	0
NJOY (279)	0	315	396	136	101	30	8	2	0
NU cig (272)	0	37	122	75	98	39	8	2	1
Ohm Gurt (187)	0	69	63	2	4	1	1	1	1
Organic Banana Nut Muffin (213)	0	48	26	-1	3	0	1	2	1
Orphan Tears (25)	0	541	142	41	41	14	4	1	0
Peach (162)	0	7	3	-2	1	-1	0	0	0
Peach Piano (14)	0	309	229	73	75	23	5	2	0
Peach Tea (139)	0	69	246	308	373	63	5	3	0
Peache Guavara (290)	0	18	50	8	18	5	2	1	0
Peaches N Cream (66)	0	119	139	28	36	13	2	1	1
Peanut Butter Cookies (3)	0	24	173	97	112	47	17	7	2
Pillow Fight (21)	0	444	214	61	61	23	5	2	0
Pina colada (169)	0	72	88	53	63	23	4	1	1
Pina colada (285)	0	17	138	145	111	39	10	3	0
Pink Lemonade (182)	0	46	212	71	63	22	5	3	2
Pixie Dust (98)	809	4296	233	106	85	26	5	2	0
PMS (177)	0	75	243	102	108	43	7	1	1
Pomegranate (134)	0	115	303	136	222	112	8	2	1
Popcorn (11)	0	42	205	80	70	25	5	1	0
Popcorn (183)	0	56	285	168	145	50	10	4	1
Poud Cake (292)	0	19	135	91	88	34	10	4	1
Pumpkin Pie (127)	0	20	285	168	161	58	15	4	1
Rainbow Candy (202)	0	1084	188	122	159	64	9	3	1
Raspberry (40)	157	275	93	18	25	8	2	0	0
Ravishing Rica Vixens (239)	0	30	169	46	51	23	17	36	79
Red Gummies (157)	0	3569	35	0	4	1	0	0	1
Red Tobacco (254)	0	122	280	81	80	31	9	3	2
Red Tobacco (281)	0	128	309	86	86	34	10	4	2
Rolling Thunder (297)	0	111	252	69	83	34	9	2	0
Root Beer (94)	0	25	151	88	110	52	17	5	1
Root Beer Float (210)	0	15	119	38	56	30	12	7	3
RY4 Classic (48)	0	59	227	86	99	43	11	3	0
RY4 Doubler (36)	0	81	286	146	127	38	7	3	0
Shamrock (217)	0	17	382	126	116	60	22	6	3
Single Malt Scotch (133)	0	82	365	66	60	20	5	2	0
Slug Juice (18)	0	173	190	85	71	18	3	2	0
Smoke Peach (277)	0	247	297	229	222	86	20	6	1
Solid Menthol (5)	0	145	357	117	110	40	9	3	2
Solstice (221)	0	1165	210	36	34	12	2	1	1
Sour Fruit Punch (118)	0	168	337	66	54	14	2	1	1
Space Jam (204)	0	238	205	97	111	54	15	5	1
Strawberries and Champagne (116)	0	163	166	57	43	13	2	0	0
Strawberry (70)	0	203	316	128	122	46	8	3	2
Strawberry Cheesecake (196)	0	56	260	58	54	22	3	1	0
Strawberry Mango Smoothie (35)	0	84	222	120	122	50	9	3	1
Strawberry Milkshake (252)	0	31	181	93	69	23	5	2	0
Strawberry Pops O'Tart (120)	0	34	165	92	110	53	16	6	3
Sub Zero Watermelon (222)	0	63	104	23	33	10	2	1	0
Sugar Cookie (93)	0	24	130	49	67	33	14	6	3
Sweet Potato Pie (77)	0	47	420	163	175	73	18	6	1
The 5th November (242)	0	87	371	164	131	56	20	8	2
The Milksman (193)	0	41	227	108	103	43	12	5	2
Tiramisu (92)	0	11	232	75	66	32	16	5	1
Tobacco (282)	0	93	379	289	295	109	25	8	2
Tobacco Mint (190)	0	131	155	45	40	13	3	1	1
Toffee (211)	0	41	232	83	104	47	11	3	0
Trip in the Woods (38)	0	37	256	107	132	60	19	7	2

Tropical Island (251)	0	76	45	11	28	8	1	1	1
True Banana (167)	0	31	129	69	149	88	11	4	1
Unicorn Blood (198)	0	31	74	46	97	63	37	3	2
USA mix (181)	0	154	267	71	75	25	7	2	0
Valkyrie (31)	0	25	188	95	117	52	16	8	3
Vamp Toes (250)	0	66	184	96	88	30	10	4	2
Vanilla (212)	0	27	234	123	114	57	29	12	4
Vanilla Bean (140)	0	14	73	22	26	11	5	3	2
Vanilla Clouds (291)	0	15	153	94	159	90	27	13	5
Vanilla Custard (51)	0	23	90	42	65	34	14	6	3
Vanilla Tobacco (8)	0	22	132	64	76	35	15	7	2
Watermelon (46)	0	234	90	11	21	8	2	0	1
White Gummies (161)	0	20	7	-4	0	-1	1	1	0
White Horse (286)	0	9	44	14	33	18	5	2	2
Wicked (278)	0	54	235	205	264	99	19	5	1
Wintergreen (52)	105	314	116	29	36	12	3	2	0
Wow (214)	0	120	219	71	63	19	8	1	0
X'smas Cake (176)	0	176	163	112	165	87	21	7	3

Table S2- Comprehensive list of E-liquids. This list includes vendor, lot number, nicotine concentration (mg/ml), PG and VG concentration (%). Unavailable information is reported as not available (N/A).

E-liquid Name	Internal reference number (IRN)	Vendor	Lot Number	Nicotine Concentration (mg/mL)	PG (%)	VG (%)
1 Leaf Tobacco	69	The Vapor Girl Inc.	114414	12	55	45
10 Leaves Tobacco	65	The Vapor Girl Inc.	114414	12	55	45
5 Leaves Tobacco	61	The Vapor Girl Inc.	114414	12	55	45
55:45 PGVG	164	Sigma-Aldrich	N/A	0	55	45
Adam's Apple	173	Adam Bomb	L1	24	NA	NA
Adam's Apple	194	Adam Bomb	L1	24	N/A	N/A
Admiral Berry Crunch	227	Mount Baker Vapor, LLC	1687534	24	50	50
Alchemy	34	The Vapor Girl Inc.	114414	12	55	45
Ambrosia	27	The Vapor Girl Inc.	114414	12	55	45
Andromeda	204	Space Jam	L1	18	N/A	N/A
Angel Lust	23	The Vapor Girl Inc.	114414	12	55	45
Anti-Venom	215	5150	L1	6	N/A	N/A
Apple	219	NOVO	L1	48	N/A	N/A
Apple	256	N/A	L1	N/A	N/A	N/A
Apple	275	N/A	L1	N/A	N/A	N/A
Apple Candy	178	Smoke Vapor Gallery	L1	24	N/A	N/A
Apple Candy	234	Visa Vapors	3.7 A1562394881	24	50	50
Apple Cinnamon	218	eVo E-liquid	2002102153	12	N/A	N/A
Apple Pie	122	The Vapor Girl Inc.	114414	12	55	45
Appletini	175	Vape Dudes	1100215617	24	50	50
Arctic Blueberry	62	The Vapor Girl Inc.	114414	12	55	45
Arctic Ice	191	Mystic Vapor Ltd.	MJO1203	6	N/A	N/A
Arctic Raspberry	78	The Vapor Girl Inc.	114414	12	55	45
Arctic Strawberry	102	The Vapor Girl Inc.	114414	12	55	45
Arctic Tobacco	68	The Vapor Girl Inc.	114414	12	55	45
Artist Liquids	241	Artists Liquids	L1	2	N/A	100
Ashes to Ashes	185	Hostile Vapes	L1	12	N/A	N/A
B&H	203	Colonial Stores	L1	N/A	N/A	100
Bad Monkey	195	Maximum Vapor Unicorn Dust	L1	18	60	40
Bada Bing!	158	E-Toxic	L1	0	55	45
Bahama Mama	45	The Vapor Girl Inc.	114414	12	55	45
Baja Burst	186	Apollo Liquid Vapor	245707979	24	N/A	N/A
Banana	114	The Vapor Girl Inc.	114414	12	55	45
Banana Nut Bread	288	Vape Wild	L1	24	50	50
Banana Nut Bread	289	N/A	L1	3	N/A	65
Banana Nut Bread Smoothie	42	The Vapor Girl Inc.	114414	12	55	45
Banana Pudding	166	The Vapor Girl Inc.	114414	0	55	45
Banana Pudding Southern Style	129	The Vapor Girl Inc.	114909	0	55	45
Banana Pudding Southern Style	165	The Vapor Girl Inc.	114414	12	55	45
Banana Yo! (Yogurt!)	63	The Vapor Girl Inc.	114414	12	55	45
Barista	180	503	L1	20	55	45
Barlett Pear	49	The Vapor Girl Inc.	114414	12	55	45
Bat Juice	249	Vampire Vape	553930	24	50	50
Battle Frogs	26	The Vapor Girl Inc.	114414	12	55	45
Bavarian Creme Donut	115	The Vapor Girl Inc.	114414	12	55	45
Berry Creamy	228	Mount Baker Vapor, LLC	L1	0	50	50
Biscotti	89	The Vapor Girl Inc.	114414	12	55	45
Black and Blue Berries	142	NJOY	Unknown	10	55	45
Black Cherry	125	The Vapor Girl Inc.	114414	12	55	45
Black Coffee	90	The Vapor Girl Inc.	114414	12	55	45
Black Dragon	20	The Vapor Girl Inc.	114414	12	55	45
Black Hawk	80	The Vapor Girl Inc.	114414	12	55	45
Black Honey Tobacco	57	The Vapor Girl Inc.	114414	12	55	45
Black Licorice	12	The Vapor Girl Inc.	114414	12	55	45
Black Peppercorn	101	The Vapor Girl Inc.	114414	12	55	45
Black Thorn	109	The Vapor Girl Inc.	114414	12	55	45
Blackberry Lemonade	53	The Vapor Girl Inc.	114414	12	55	45
Blackberry Panda	44	The Vapor Girl Inc.	114414	12	55	45
Blood Orange	141	NJOY	A4F32	10	55	45
BLU Refill Tobacco	270	N/A	L1	N/A	N/A	N/A
Blue Cherry	257	N/A	L1	N/A	N/A	N/A
Blue Classic Tobacco	262	N/A	L1	N/A	N/A	N/A
Blue DUDE	59	The Vapor Girl Inc.	114414	12	55	45
Blue Mist	276	N/A	L1	N/A	N/A	N/A
Blue Moo	106	The Vapor Girl Inc.	114414	12	55	45

Blue Pom (Pomegranate)	76	The Vapor Girl Inc.	114414	12	55	45
Blueberry	207	V e-liquid	L1	0	N/A	N/A
Blueberry Cinnamon-Streusal Muffin	100	The Vapor Girl Inc.	114414	12	55	45
Blueberry Lemonade	55	The Vapor Girl Inc.	114414	12	55	45
Blueberry Tobacco	39	The Vapor Girl Inc.	114414	12	55	45
Briar Patch	67	The Vapor Girl Inc.	114414	12	55	45
Brittany's Blue Blend	50	The Vapor Girl Inc.	114414	12	55	45
Bubble Gum	56	The Vapor Girl Inc.	114414	12	55	45
Bubbly Berry	79	The Vapor Girl Inc.	114414	12	55	45
Butter Crunch	135	NJOY	A4727	10	55	45
Butter Pecan	220	Kobra Juice	L1	18	30	70
Butterscotch	112	The Vapor Girl Inc.	114414	12	55	45
Buttery Nipple	96	The Vapor Girl Inc.	114414	12	55	45
Cake Batter	295	Sweet Vapes	124	24	N/A	N/A
Candy Cane	60	The Vapor Girl Inc.	114414	12	55	45
Candy Corn	43	The Vapor Girl Inc.	114414	12	55	45
Cappuccino	201	Joy E Tech	L1	12	N/A	N/A
Cappuchino	184	EV Cigarettes	L1	24	50	50
Captain Suckle	19	The Vapor Girl Inc.	114414	12	55	45
Captain Zack	47	The Vapor Girl Inc.	114414	12	55	45
Captain Zack Cigar	128	The Vapor Girl Inc.	1523673	0	55	45
Caramel	188	Hangsen	L1	24	N/A	N/A
Caramel Apple	111	The Vapor Girl Inc.	114414	12	55	45
Caramel Corn Crunch	64	The Vapor Girl Inc.	114414	12	55	45
Carnage	171	ANML	L1	12	NA	NA
Cat Nip	105	The Vapor Girl Inc.	114414	12	55	45
Chai	84	The Vapor Girl Inc.	114414	12	55	45
Chai Latte	85	The Vapor Girl Inc.	114414	12	55	45
Chai Tea	216	Coval Vapes	6844	18	30	70
Cheesecake	179	Tasty Vapor	L1	36	N/A	N/A
Cheesecake with Graham Cracker Cru	97	The Vapor Girl Inc.	114414	12	55	45
Cherry	274	N/A	L1	N/A	N/A	N/A
Cherry Kola	16	The Vapor Girl Inc.	114414	12	55	45
Chicken and Waffles	37	The Vapor Girl Inc.	114414	12	55	45
Chocolate Banana	87	The Vapor Girl Inc.	114414	12	55	45
Chocolate Covered Raisins	54	The Vapor Girl Inc.	114414	12	55	45
Chocolate Dipt Raspberries	104	The Vapor Girl Inc.	114414	12	55	45
Chocolate Fudge	82	The Vapor Girl Inc.	114414	12	55	45
Chocolate Moo	74	The Vapor Girl Inc.	114414	12	55	45
Chocolate Pecan Fudge	95	The Vapor Girl Inc.	114414	12	55	45
Chocolate Tobacco Heaven	126	The Vapor Girl Inc.	114414	12	55	45
Cig	273	N/A	L1	N/A	N/A	N/A
Cinnamon	294	Heartland Vapes	150911001	24	20	80
Cinnamon Roll	15	The Vapor Girl Inc.	114414	12	55	45
Cinnamon Roll	192	Pack Juice	L1	24	N/A	N/A
Cinn-Full Pear	300	Provari Fuel	5121L	24	70	30
Cinn-Full Pear	301	Provari Fuel	5019K	24	N/A	N/A
Circus Guava	75	The Vapor Girl Inc.	114414	12	55	45
City of Angels	163	E-Toxic	L1	0	55	45
Classic Tobacco	136	NJOY	A4G03	10	55	45
Classics Cookie Monster	229	Phoenix Vapor Shop	L1	18	N/A	N/A
Classics Cookie Monster	230	Phoenix Vapor Shop	L1	6	N/A	N/A
Cloud Candy	200	Cloud V E-Juice	L1	11	30	70
Clove	172	Vaping zone	L1	36	NA	NA
Clove Cigar	123	The Vapor Girl Inc.	114414	12	55	45
Coconut Rum	107	The Vapor Girl Inc.	114414	12	55	45
Coconut Water	72	The Vapor Girl Inc.	114414	12	55	45
Cola	287	N/A	L1	N/A	N/A	N/A
Comb The Desert	199	The Schwartz E-Liquid	L1	6	N/A	N/A
Cool Mint	124	The Vapor Girl Inc.	114414	12	55	45
Cotton Berry	108	The Vapor Girl Inc.	114414	12	55	45
Cotton Candy	197	Vapor + HQ	150619001	12	50	50
Crabtastic!	24	The Vapor Girl Inc.	114414	12	55	45
Cranberry Crunch	117	The Vapor Girl Inc.	114414	12	55	45
Cranberry Delight!	99	The Vapor Girl Inc.	114414	12	55	45
Crispy Melon	121	The Vapor Girl Inc.	114414	12	55	45
Cuba Libre	30	The Vapor Girl Inc.	114414	12	55	45
DB's Dessert	58	The Vapor Girl Inc.	114414	12	55	45
Death Flirt	28	The Vapor Girl Inc.	114414	12	55	45
Desert Cow	22	The Vapor Girl Inc.	114414	12	55	45
Devil's Cut	170	Hard Cocktails	080415-1	NA	NA	NA
Double Espresso	137	NJOY	A4F40	10	55	45

Dragon's Blood	247	EC Blend	78059	36	50	50
Dragon's Tear	246	EC Blend	62556	0	50	50
Drunk Monkey	209	G2 Vapor	254031815	18	N/A	100
Dulce de Leche	73	The Vapor Girl Inc.	114414	12	55	45
Energon	13	The Vapor Girl Inc.	114414	12	55	45
Fab 5	268	N/A	L1	N/A	N/A	N/A
French Vanilla Cinnamon Coffee	83	The Vapor Girl Inc.	114414	12	55	45
French Vanilla Hazelnut Espresso	86	The Vapor Girl Inc.	114414	12	55	45
Fruit Loopy	41	The Vapor Girl Inc.	114414	12	55	45
Fruity Juice	233	Visa Vapors	A64.67.1.1	0	50	50
Georgia Juicy Peach	33	The Vapor Girl Inc.	114414	12	55	45
GG	261	N/A	L1	N/A	N/A	N/A
Glazed Doughnut	244	Sirius Vapors	1505191	18	40	60
Gob-Stopper	232	Vaper Crue	L1	24	N/A	N/A
Grandmaster	296	Five Pawns	110	18	50	50
Grape Soda	103	The Vapor Girl Inc.	114414	12	55	45
Grape!	32	The Vapor Girl Inc.	114414	12	55	45
Green Gummies	159	E-Toxic	L1	0	55	45
Gummy	223	Vapor Fi	30-70-36 VF 274493	36	70	30
Hawaiian Punch	224	Vapor World	L1	24	N/A	N/A
Heavy Hitters	205	Clouds Vape	L1	12	N/A	N/A
Honey Vanilla Tobacco	119	The Vapor Girl Inc.	114414	12	55	45
Hot Cinnamon Candies	4	The Vapor Girl Inc.	1523673	12	55	45
Hot Cinnamon Candies	130	The Vapor Girl Inc.	114909	0	55	45
I Love Donuts	226	Mad Hatter Juice	L1	12	40	60
Icy Blast	81	The Vapor Girl Inc.	114414	12	55	45
Jay's A Straw From Heaven	208	Jay's Straw from Heaven	L1	18	N/A	100
Key Lime Pie	91	The Vapor Girl Inc.	114414	12	55	45
KIWI Blast	113	The Vapor Girl Inc.	114414	12	55	45
Kola	2	The Vapor Girl Inc.	114414	12	55	45
Kola No Nicotine	131	The Vapor Girl Inc.	114909	0	55	45
Lemon Meringue Pie	110	The Vapor Girl Inc.	114414	12	55	45
Lock Jaw	235	The Vapor Emporium	L1	24	N/A	N/A
Looper	225	ANML	L1	3	N/A	N/A
Looper	237	ANML	L1	12	N/A	N/A
Magic Dragon	174	Fantasia E-liquid Flavoring	L1	N/A	NA	NA
Marc's Burro Tobacco	17	The Vapor Girl Inc.	114414	12	55	45
Marshmallow	71	The Vapor Girl Inc.	114414	12	55	45
Menthol	138	NJOY	A4F44	10	55	45
Menthol	253	N/A	L1	N/A	N/A	N/A
Menthol	255	N/A	L1	N/A	N/A	N/A
Menthol Tobacco	10	The Vapor Girl Inc.	114414	12	55	45
Menthol Tobacco	132	The Vapor Girl Inc.	114909	0	55	45
Mint	269	N/A	L1	N/A	N/A	N/A
Missed Her Cookie	88	The Vapor Girl Inc.	114414	12	55	45
Mochachino	293	HiliQ	L1	18	N/A	N/A
Mojito	160	E-Toxic	L1	0	55	45
Mt. DUDE	29	The Vapor Girl Inc.	114414	12	55	45
N/A	263	N/A	L1	N/A	N/A	N/A
Nana Banana	189	Vapor Shark	9873239	24	N/A	N/A
Nebula	168	Ben Johnson's eJuice	NB-18-0003	18	NA	NA
Night Time's Blues	231	Vaper Crue	L1	24	N/A	N/A
NJOY	258	N/A	L1	N/A	N/A	N/A
NJOY	259	N/A	L1	N/A	N/A	N/A
NJOY	279	N/A	L1	N/A	N/A	N/A
NU cig	272	N/A	L1	N/A	N/A	N/A
Ohm Gurt	187	Juicy Ohms	L1	3	N/A	N/A
Organic Banana Nut Muffin	213	Virgin Vapor	80114	0	N/A	100
Orphan Tears	25	The Vapor Girl Inc.	114414	12	55	45
Peach	162	E-Toxic	L1	0	55	45
Peach Piano	14	The Vapor Girl Inc.	114414	12	55	45
Peach Tea	139	NJOY	A4F35	10	55	45
Peache Guavara	290	N/A	L1	3	N/A	65
Peaches N Cream	66	The Vapor Girl Inc.	114414	12	55	45
Peanut Butter Cookies	3	The Vapor Girl Inc.	114414	12	55	45
Pillow Fight	21	The Vapor Girl Inc.	114414	12	55	45
Pina Colada	169	Mister-E-Liquid	L1	24	NA	33
Pina Colada	285	N/A	L1	N/A	N/A	N/A
Pink Lemonade	182	Element E-Liquid	L864	3	20	80
Pixie Dust	98	The Vapor Girl Inc.	114414	12	55	45
PMS	177	Liberty Vapor Vape or Dye	918-266-4716	24	70	30
Pomegranate	134	NJOY	4AF34	10	55	45

Popcorn	11	The Vapor Girl Inc.	114414	12	55	45
Popcorn	183	Joy E Tech	L1	12	N/A	N/A
Pound Cake	292	Glas	L1	12	N/A	N/A
Product-X	206	Bombies	L1	12	N/A	100
Pumpkin Pie	127	The Vapor Girl Inc.	114414	12	55	45
Rainbow Candy	202	MNG Vape	571	24	N/A	N/A
Raspberry	40	The Vapor Girl Inc.	114414	12	55	45
Ravishing Rica Vixens	239	Undead Vapes	L1	24	N/A	N/A
Red Gummies	157	E-Toxic	L1	0	55	45
Red Tobacco	254	N/A	L1	N/A	N/A	N/A
Red Tobacco	281	N/A	L1	N/A	N/A	N/A
Rolling Thunder	297	Lightning Vapes	6	6	N/A	N/A
Root Beer	94	The Vapor Girl Inc.	114414	12	55	45
Root Beer Float	210	Vapor Beast Brew	L1	24	50	50
RY4 Classic	48	The Vapor Girl Inc.	114414	12	55	45
RY4 Doubler	36	The Vapor Girl Inc.	114414	12	55	45
Shamrock	217	Halo	Sham1001251	12	N/A	N/A
Single Malt Scotch	133	NJOY	4AF42	10	55	45
Slug Juice	18	The Vapor Girl Inc.	114414	12	55	45
Smoke Peach	277	N/A	L1	N/A	N/A	N/A
Solid Menthol	5	The Vapor Girl Inc.	114414	12	55	45
Solstice	221	Space Jam	1019015	12	N/A	N/A
Sour Fruit Punch	118	The Vapor Girl Inc.	114414	12	55	45
Strawberries and Champagne	116	The Vapor Girl Inc.	114414	12	55	45
Strawberry	70	The Vapor Girl Inc.	114414	12	55	45
Strawberry Cheesecake	196	The Real	SC12052015	12	N/A	N/A
Strawberry Mango Smoothie	35	The Vapor Girl Inc.	114414	12	55	45
Strawberry Milkshake	252	Vampire Vape	553957	24	60	40
Strawberry Pops O'Tart	120	The Vapor Girl Inc.	114414	12	55	45
Sub Zero Watermelon	222	Kidney Puncher	L1	18	50	50
Sugar Cookie	93	The Vapor Girl Inc.	114414	12	55	45
Sweet Potato Pie	77	The Vapor Girl Inc.	114414	12	55	45
The 5th of November	242	Illpluminati	L1	6	N/A	N/A
The Milkman	193	The Milkman	BOTO 278	6	N/A	99
Tiramisu	92	The Vapor Girl Inc.	114414	12	55	45
Tobacco	282	N/A	L1	N/A	N/A	N/A
Tobacco Mint	190	UK E-Cig Store	614	24	70	30
Toffee	211	Jac Vapour	2416	24	80	20
Trip in the Woods	38	The Vapor Girl Inc.	114414	12	55	45
Tropical Island	251	Vampire Vape	553959	24	60	40
True Banana	167	TS Vapes	L1	36	NA	NA
Unicorn Blood	198	Fuzion Vapor	03051UB15120	12	N/A	N/A
USA Mix	181	Dekang	L1	18	N/A	N/A
Valkyrie	31	The Vapor Girl Inc.	114414	12	55	45
Vamp Toes	250	Vampire Vape	554361	24	60	40
Vanilla	212	Joy E Tech	L1	18	N/A	N/A
Vanilla Bean	140	NJOY	A4F33	10	55	45
Vanilla Clouds	291	N/A	L1	24	50	50
Vanilla Custard	51	The Vapor Girl Inc.	114414	12	55	45
Vanilla Tobacco	8	The Vapor Girl Inc.	114414	12	55	45
Watermelon	46	The Vapor Girl Inc.	114414	12	55	45
White Gummies	161	E-Toxic	L1	0	55	45
White Horse	286	N/A	L1	N/A	N/A	N/A
Wicked	278	N/A	L1	N/A	N/A	N/A
Wintergreen	52	The Vapor Girl Inc.	114414	12	55	45
Wow	214	Wow	L1	N/A	N/A	N/A
Xmas Cake	176	Holiday Hangsen E-liquids	200-193-3	24	N/A	N/A
